# LDH isozymes as targets for cancer therapy

**DOI:** 10.1080/14756366.2026.2639168

**Published:** 2026-03-16

**Authors:** Yiqian Hou, Yanying Zhao, Qinghua He, Meilin Wang, Qinglian Zhang

**Affiliations:** ^a^ Key Laboratory of Qinghai–Tibetan Plateau Animal Genetic Resource Reservation and Utilization, Southwest Minzu University, Chengdu, People’s Republic of China; ^b^ School of Laboratory Medicine, Chengdu Medical College, Chengdu, People’s Republic of China

**Keywords:** Cancer therapy, glycolysis, LDH isozymes, Warburg effect

## Abstract

The Warburg effect, a hallmark of cancer, positions lactate dehydrogenase (LDH) as a key therapeutic target. Mammals possess three LDH isozymes (LDH-A, LDH-B, LDH-C) with distinct properties. This review critically re-evaluates the simplistic ‘aerobic-anaerobic’ paradigm, emphasizing that all isozymes catalyze reversible pyruvate-lactate conversion and contribute to tumor metabolism in a context-dependent manner. While LDH-A inhibition is a primary focus, challenges include metabolic plasticity and compensatory LDH-B upregulation. LDH-B plays a critical role in mitochondrial lactate oxidation. We highlight LDH-C as a compelling cancer/testis antigen target. Beyond glycolysis, LDH-C exhibits unique substrate promiscuity, generating oncometabolites like s-2-hydroxyglutarate from α-ketoglutarate. Its structural distinctions and restricted normal tissue expression offer opportunities for highly selective therapy. A comprehensive understanding of all three isozymes is essential for developing effective metabolic interventions against cancer.

## Graphical Abstract

**Figure:**
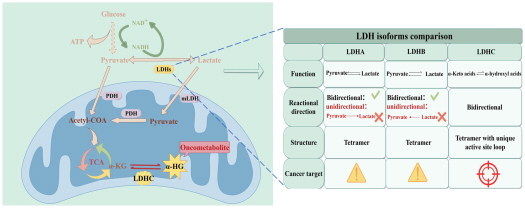

## 1. LDH: a central driver of the Warburg effect in cancer

Highly active glycolysis is a distinct biochemical phenotype of cancer cells, persisting even in the presence of oxygen—a phenomenon termed the Warburg effect. Glycolysis not only provides a crucial energy source but also generates intermediate metabolites essential for synthesising amino acids, lipids, and other components required for cancer cell proliferation. Furthermore, lactate, the end product of glycolysis, functions as an indirect epigenetic regulator in cancer cells^1^^,^^2^. Consequently, inhibiting glycolysis represents a significant strategy for combating cancer^3^.

**Figure 1.**
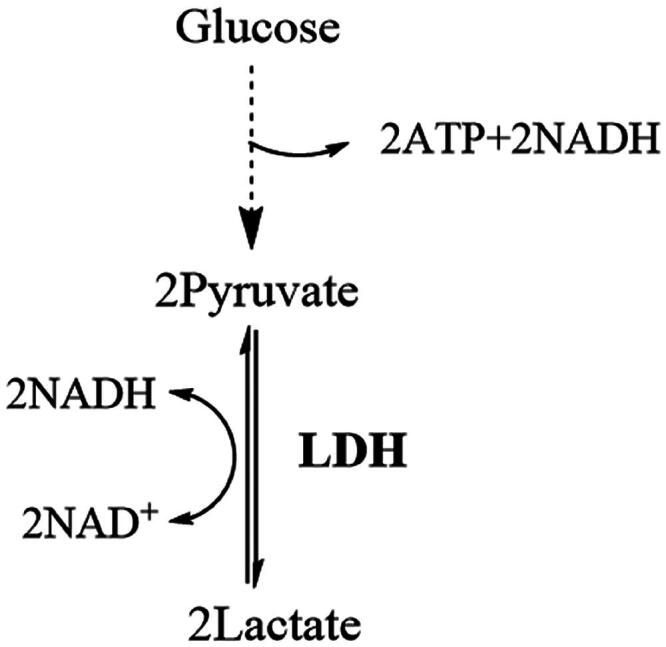
Simplified diagram of the glycolysis pathway, dotted arrow indicates multi-step catalytic processes, LDH is in bold and is at the end of the pathway.

LDH is a key enzyme positioned at the terminus of the glycolytic pathway (Figure 1). It catalyses the reduction of pyruvate to lactate while oxidising NADH to NAD^+^. The regeneration of NAD^+^ enables glycolysis to continue under anaerobic conditions. Therefore, LDH is considered an attractive target for anticancer research aimed at inhibiting glycolysis^4^.

## 2. Phylogeny, distinct tissue distribution, and catalytic properties of LDH isozymes

**Figure 2.**
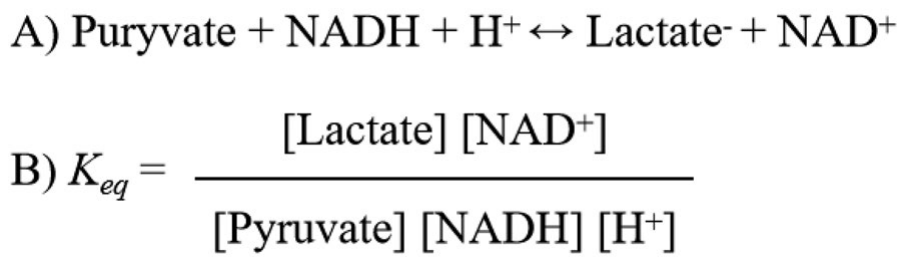
A) Chemical reaction formula catalysed by LDH; B) Equilibrium constant (*K_eq_*) calculation formula.

LDH is an NAD^+^-dependent tetrameric enzyme primarily catalysing the interconversion of pyruvate and lactate (Figure 2A). Mammals possess three types of LDH subunits: LDH-A, LDH-B, and LDH-C, which can assemble into six distinct tetrameric isozymes. The A and B subunits form five isozymes: LDH-A4, LDH-A3B1, LDH-A2B2, LDH-A1B3, and LDH-B4. The C subunit forms only one isozyme: LDH-C4^5^^,^^6^. The primary differences between these subunits are summarised in Table 1.

**Table 1.** Comparative characteristics of LDH isozymes in physiology and cancer biology.

Characteristic	LDH-A	LDH-B	LDH-C
Tissue distribution	Predominantly glycolytic tissues (e.g., skeletal muscle)^7^	Predominantly oxidative tissues (e.g., heart muscle)^8^^,^^9^	Restricted expression (germ cells)^10^^,^^11^; Cancer-testis antigen (CTA)^12^
Catalytic properties	Bidirectional conversion between pyruvate and lactate^13^^,^^14^	Bidirectional conversion between pyruvate and lactate^13^^,^^14^	Bidirectional conversion between α-keto acids and α-hydroxyl acids (substrate promiscuity)^15^
Kinetic features	Lower affinity for pyruvate and lactate than LDH-B; more tolerant to substrate inhibition^13^^,^^14^	Higher affinity for pyruvate and lactate than LDH-A^13^^,^^14^; less tolerant to substrate inhibition^8^	Highest affinity for pyruvate among the three isoforms^15^
Protein structure	Tetramer^5^	Tetramer^5^	Tetramer with distinct active site loop compare to LDH-A and LDH-B^5^
Expression in cancer	Upregulated across many cancers^16–18^	Upregulated in specific subsets^19^	Aberrant re-expression in some cancers^15^^,^^20^
Roles in cancer	Drives glycolysis^21^, proliferation^4^, and invasion^22^	Drives glycolysis, promotes immune evasion^23^, lactate oxidation^24^	Drives glycolysis, metabolism reprogramming^25^, maintain genomic integrity^26^
Therapeutic status	Major target; several small-molecule inhibitors in clinical trials^27^^,^^28^	Emerging target; lack specific inhibitors over LDH-A^23^	Attractive CTA target; more amenable to selective inhibition^15^^,^^20^

### 2.1. Phylogeny of the three LDH isoforms

In mammals, the core lactate dehydrogenase A (LDHA) and lactate dehydrogenase B (LDHB) genes represent the ancestral LDH lineages maintained across vertebrates. Comparative genomic and phylogenetic analyses of human, mouse, opossum and platypus show that these two loci are conserved among diverse mammalian clades, including monotremes, marsupials, and eutherians^29^. Throughout vertebrate evolution, LDHA and LDHB have maintained distinct biochemical properties and tissue-biased expression patterns.

In addition to LDHA and LDHB, many mammals possess a third somatic-type gene, Lactate dehydrogenase C (LDHC), which originated via a duplication of the LDHA locus early in mammalian evolution^29^^,^^30^. Following its emergence, LDHC rapidly evolved under positive selection, diverging in both sequence and regulatory control to support sperm-specific energy metabolism. Experimental evidence from mouse knockout studies shows that LDHC accounts for most LDH activity in sperm and is indispensable for sperm motility and male fertility, illustrating how gene duplication enabled functional specialisation^11^.

### 2.2. Tissue-specific expression profiles

Early studies identified the LDH-A subunit as predominantly expressed in skeletal muscle, while the LDH-B subunit is primarily expressed in cardiac muscle. This observation led to the widely accepted “aerobic–anaerobic” theory, proposing that LDH-A is suited to tissues experiencing periodic oxygen deprivation (e.g., skeletal muscle) and converts pyruvate to lactate, whereas LDH-B is adapted for aerobic metabolism (e.g., cardiac muscle) and converts lactate to pyruvate^7^.

However, this tissue-distribution-based theory conflicts with the biochemical nature of LDH and modern physiological evidence. Biochemically, LDH catalyses a reversible, near-equilibrium reaction. At physiological pH (7.4) and temperature (37 °C), the equilibrium constant is exceedingly high (*K*_eq_ = 1.62 × 10^11^ M^−1^), strongly favouring lactate formation^31^ (Figure 2B). Due to the high intracellular concentration and activity of LDH, the reaction likely operates near equilibrium *in vivo*^18^. This means the direction of net metabolic flux is not dictated by the isozyme type but is governed by local metabolite concentrations, redox state (NAD^+^/NADH), and upstream pathway flux^32^^,^^33^. Computational analyses also show that altering the LDH-A:LDH-B ratio has minimal impact on steady-state lactate levels compared to changes in total LDH abundance or upstream drivers. Furthermore, the rigid functional dichotomy is contradicted by the fact that highly aerobic tissues like the heart and brain dynamically both produce and oxidise lactate^9^. These observations support a contemporary metabolic framework where lactate is a central, aerobically produced metabolite involved in intercellular shuttling and serves as a major oxidative fuel^9^.

Despite this evidence, many researchers maintain that LDH-A mainly catalyses the reduction of pyruvate to lactate, while LDH-B primarily facilitates the oxidation of lactate to pyruvate. Consequently, LDH-A is often viewed as a more attractive anticancer target^34–37^ than LDH-B, which is sometimes considered a lactate scavenger^38^. LDH-C is specifically expressed in testicular tissue, appearing only in specific germ cells during spermatogenesis and not in somatic cells. However, it is aberrantly expressed in certain cancer cells and is thus classified as a cancer/testis antigen (CTA)^39^.

### 2.3. Kinetic divergence and substrate specificity across isozymes

The three LDH isozymes exhibit distinct enzymatic characteristics. LDH-A has a lower affinity (higher *K_m_*) for both pyruvate and lactate compared to LDH-B^13^^,^^14^. LDH-A4 is also more tolerant than LDH-B4 to substrate and product inhibition. The inhibition constant (*Ki*) for l-lactate is 130 mM for LDH-A4 and 26.0 mM for LDH-B4. The *K*_i_ for pyruvate inhibition is 0.28 mM for LDH-A4 and 0.18 mM for LDH-B4^8^.

It is important to note that some publications incorrectly state that LDH-A exhibits high affinity for pyruvate (facilitating pyruvate-to-lactate conversion), while LDH-B exhibits high affinity for lactate (facilitating lactate-to-pyruvate conversion)^40^^,^^41^. This is misleading; LDH-B actually demonstrates higher affinity for both pyruvate and lactate than LDH-A. The confusion may stem from misinterpreting the Michaelis constant (*K*_m_), where a higher *K*_m_ indicates lower affinity. Another study suggests that pyruvate inhibits LDH-B more potently than lactate, thereby favouring lactate-to-pyruvate conversion^38^. However, this ignores the fact that both pyruvate and lactate inhibit LDH-B more strongly than LDH-A^8^.

While cytosolic pyruvate and lactate concentrations are not extensively documented, cells with active glycolysis generally exhibit higher levels of both metabolites compared to those relying on oxidative phosphorylation^42^. Evolutionarily, LDH-A appears adapted to environments with elevated pyruvate and lactate concentrations, whereas LDH-B may be suited to conditions with lower substrate levels^43^. This specialisation likely supports efficient attainment of reaction equilibrium under varying metabolic demands.

Among the LDH isozymes, LDH-C not only exhibits the highest affinity and catalytic efficiency for pyruvate but also a unique substrate promiscuity, efficiently reducing α-keto acids with extended aliphatic chains like α-ketoglutarate (α-KG) to their corresponding α-hydroxyl acids^15^. The overall structures of the A, B, and C subunits are highly similar. However, the conformation of the substrate-binding loop in LDH-C is markedly more open compared to LDH-A and LDH-B. This structural feature correlates with its enhanced ability to catalyse reactions involving α-keto acids with longer fatty chains^15^.

## 3. LDH-A as target: promise and limitations

LDH-A is overexpressed in various cancer types, contributing to processes critical for cancer progression. Elevated LDH-A expression promotes epithelial-mesenchymal transition (EMT) in lung adenocarcinoma and renal cell carcinoma^44^^,^^45^, drives angiogenesis in endometrial cancer^46^, and enhances invasion and migration in breast and gastric cancer cells^22^^,^^47^. Numerous studies have shown that silencing LDH-A expression or pharmacologically inhibiting its activity with small molecules markedly suppresses cancer cell proliferation^4^^,^^16^^,^^21^^,^^48^^,^^49^. In multiple mouse models, including xenograft and genetically engineered models, genetic ablation or inhibition of LDH-A markedly reduces tumour growth across various cancer types, such as lung cancer^16^^,^^17^, hepatocellular carcinoma^18^, and pancreatic and colorectal cancers^50^, thereby establishing LDH-A as a promising therapeutic target.

The primary mechanism of LDH-A inhibition involves suppressing glycolysis, thereby limiting the energy supply to cancer cells. Under normoxic conditions, inhibiting glycolysis can shift cancer cells towards oxidative phosphorylation (OXPHOS), increasing reactive oxygen species (ROS) levels and DNA damage, potentially inducing apoptosis. Under hypoxic conditions, however, the restricted availability of alternative energy sources may critically impair proliferation^4^^,^^21^. Additionally, glycolytic intermediates serve as essential precursors for anabolic processes supporting cancer cell growth^51^. Glycolysis also generates H^+^, creating an acidic microenvironment that promotes cancer development, progression, and immune evasion^52^.

Individuals with LDH-A gene deficiencies lack functional LDH-A protein. While they experience muscle stiffness and myoglobinuria following intense exercise, they remain asymptomatic under normal conditions^27^^,^^53^. This observation highlights LDH-A’s potential as an effective therapeutic target with a reduced likelihood of severe adverse effects from its inhibition. Developing selective small-molecule inhibitors for LDH-A is an active area of research, with several candidates advancing to clinical trials (details beyond this article’s scope; see recent reviews^27^^,^^28^).

Despite its promise, targeting LDH-A faces limitations: (1) The high structural similarity between LDH-A and LDH-B poses a significant challenge for developing selective LDH-A inhibitors^54^. Many inhibitors identified preclinically lack specificity, and concurrent inhibition of both isozymes may cause adverse effects. (2) Cancer cells may upregulate LDH-B to compensate for LDH-A inhibition, reducing therapeutic efficacy^18^. (3) Cancer cells exhibit metabolic plasticity; blocking glycolysis may trigger upregulation of alternative pathways like glutamine metabolism or fatty acid oxidation to sustain energy production and survival^55^^,^^56^.

## 4. LDH-B: compensatory expression, immunoregulatory function, and mitochondrial lactate oxidation

LDH-B has received less attention than LDH-A, primarily due to the assumption that LDH-A catalyses pyruvate-to-lactate conversion while LDH-B facilitates the reverse reaction. However, recent studies indicate LDH-B is also highly expressed in certain cancers, including triple-negative breast cancer^19^, lung cancer^57^, and colon cancer stem cells^58^. Notably, inhibiting LDH-A alone can induce compensatory LDH-B upregulation^18^. Only simultaneous knockout of both LDH-A and LDH-B effectively abolishes glycolysis in cancer cells^59^, underscoring LDH-B’s role in glycolytic maintenance and cancer progression^60^. *In vivo* studies show that LDH-B promotes tumour growth through non-canonical metabolic mechanisms. For instance, in mouse xenograft models, LDH-B sustains tumour-initiating cells in non-small-cell lung cancer by supporting mitochondrial metabolism and nucleotide biosynthesis^23^. Consistently, depleting LDH-B suppresses tumour growth by disrupting redox balance and glutathione metabolism, thereby increasing oxidative stress^61^. Regarding immunosuppression, LDH-A and LDH-B operate through distinct pathways. LDH-A drives immune evasion primarily via lactate production, which acidifies the tumour microenvironment, suppresses CD8^+^ T and NK cells, promotes immunosuppressive cell populations, and upregulates PD-L1^62^. In contrast, LDH-B employs a lactate-independent mechanism, disrupting redox homeostasis in CD8^+^ T cells. Recent work demonstrates that LDH-B inhibits G6PD, reduces NADPH availability, and triggers redox-stress–mediated T-cell dysfunction and disulfidptosis, thereby undermining antitumor immunity^63^.

**Figure 3.**
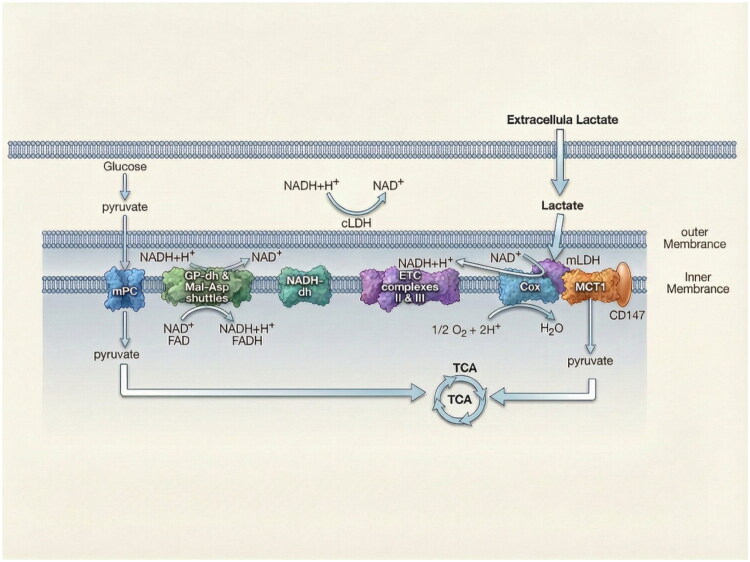
The intracellular lactate shuttle theory. The lactate shuttle theory explains how lactate functions as an important fuel source. In this process, lactate originating from intracellular glycolysis or from extracellular sources, can be transported into mitochondria and oxidised back to pyruvate for energy production. The mitochondrial lactate oxidation complex (mLOC) is a protein assembly located on the inner mitochondrial membrane that facilitates this process. The mLOC is composed of mLDH, mMCT1, and COx, with basigin (CD147) acting as a scaffold protein. In this system, mLDH oxidises lactate to pyruvate in coordination with COx, and the resulting pyruvate is transported into the mitochondrial matrix via mMCT1. The endergonic lactate oxidation is coupled with the exergonic redox reactions of COx, enabling the reaction to proceed efficiently. This pathway allows cells to efficiently utilise lactate as a major energy source. Adapted from Brooks et al.’s review article^65^.

Chen et al. reported that mitochondrial LDH (mLDH) in cancer cells predominantly consists of LDH-B, identified using anti-LDH-B antibodies and transmission electron microscopy^24^. mLDH is a key component of the mitochondrial lactate oxidation complex (mLOC), located on the inner mitochondrial membrane. The mLOC comprises mLDH, mitochondrial monocarboxylate transporter 1 (mMCT1), cytochrome oxidase (COx), and the transmembrane glycoprotein basigin (CD147), as described in the intracellular lactate shuttle theory. According to this model, lactate produced in the cytoplasm or imported from the extracellular space diffuses into the mitochondrial intermembrane space, where mLOC oxidises it to pyruvate. Pyruvate is then transported into the mitochondrial matrix. This energy-dependent lactate oxidation is coupled to the energy-releasing redox reactions of COx (Figure 3)^9^. Within the matrix, pyruvate undergoes further oxidation to acetyl coenzyme A (acetyl-CoA), which enters the tricarboxylic acid (TCA) cycle to produce energy or synthesise lipids, amino acids, and other metabolites^9^. Chen et al. also demonstrated that both ^13^C and 2-^2^H labels from isotopically enriched lactate incorporate into mitochondrial metabolites, including lipids. Deng et al. later showed that targeted LDH-B knockout significantly reduced mitochondrial metabolite levels and OXPHOS, impairing the viability of tumour-initiating cells^23^. Collectively, these findings support the lactate shuttle theory and highlight LDH-B’s critical role in mitochondrial metabolism and cancer cell survival.

Notably, work by Brooks et al. suggests both LDH-B and LDH-A may be present within mitochondria^64^. Given the high sequence similarity between LDH-A and LDH-B, antibodies targeting LDH-B could potentially cross-react. Therefore, further studies are needed to definitively identify the specific isozyme(s) constituting mLDH and clarify its role in mitochondrial function.

## 5. LDH-C: an emerging cancer/testis antigen with oncogenic catalytic versatility

LDH-C is a highly conserved, testis-specific gene expressed exclusively in the testes of marsupials and mammals. The protein is abundant in sperm, accounting for ∼82% of total LDH catalytic activity^11^. This high conservation reflects significant evolutionary selective pressure. LDH-C is crucial for sperm aerobic glycolysis, enabling adaptation to the microenvironment. Aerobic glycolysis is the primary pathway for sperm capacitation, reducing oxygen dependence and enhancing function in the hypoxic female reproductive tract. This metabolic strategy also minimises ROS production, protecting sperm DNA from oxidative damage^66^^,^^67^. It is noteworthy that sperm also contain LDH-A, tightly associated with the fibrous sheath^68^.

In normal human tissues, LDH-C expression is restricted to specific germ cells during spermatogenesis, first appearing in primary spermatocytes and significantly increasing after meiosis, particularly in spermatids and mature spermatozoa^10^^,^^69^^,^^70^. Koslowski reported that besides testicular expression, LDH-C is widely expressed in cancers, notably in lung cancer (47%), melanoma (44%), and breast cancer (35%)^39^. Consequently, LDH-C is classified as a CTA. Elevated LDH-C expression correlates with poor prognosis in hepatocellular carcinoma, lung cancer, and breast cancer. Serum or exosomal LDH-C has been proposed as a biomarker for evaluating therapeutic efficacy^12^^,^^20^^,^^71^. Functionally, LDH-C overexpression enhances proliferation, metastatic potential in lung cancer cells, and accelerates xenograft tumour growth, while silencing LDH-C inhibits proliferation^15^^,^^20^^,^^72^.

LDH-C contributes to cancer progression through mechanisms beyond energy provision via glycolysis. Emerging evidence implicates LDH-C in regulating the DNA damage response (DDR) and supporting long-term survival of breast cancer cell^72^. Silencing LDH-C induces giant cell formation, increases nuclear aberrations, and destabilises microtubules. Molecularly, this disrupts cell cycle checkpoints, leading to DNA damage accumulation, ultimately driving mitotic catastrophe and cell death^72^.

**Figure 4.**
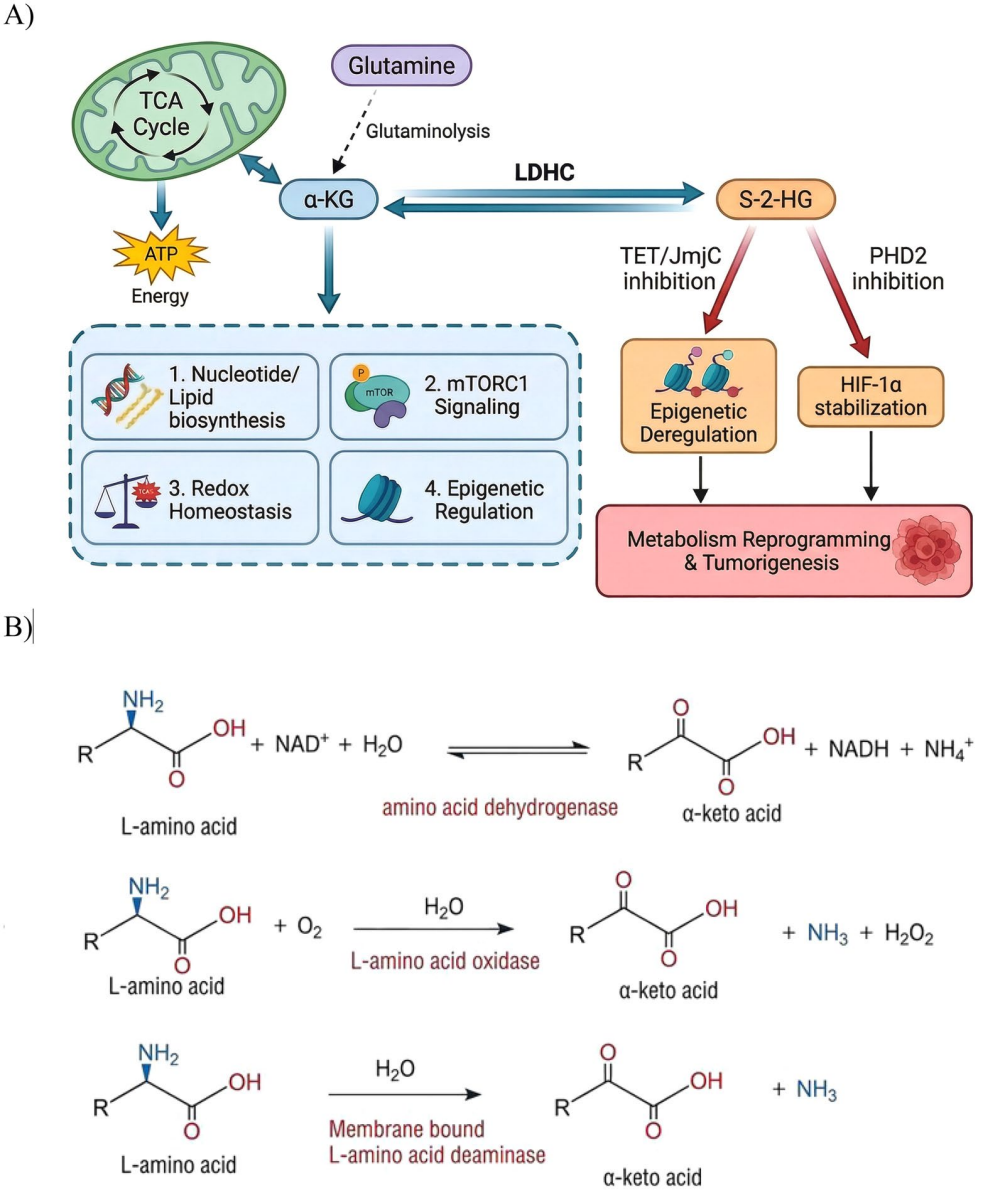
(A) Roles of α-KG and S-2-HG in cellular processes, α-KG, derived from the tricarboxylic acid (TCA) cycle and glutamine metabolism, supports multiple cellular processes: 1) Nucleotide and lipid biosynthesis, 2) mTORC1-mediated anabolic signalling, 3) redox homeostasis maintenance, and 4) epigenetic regulation via chromatin-modifying enzymes. S-2-HG, an oncometabolite generated by LDHC-mediated reduction of α-KG, drives tumorigenesis via two pathways: 1) Epigenetic dysregulation: competitive inhibition of TET dioxygenases and JmjC-domain histone demethylases, blocking DNA/histone demethylation; 2) HIF-1α stabilisation: inhibition of prolyl hydroxylases (PHDs), preventing HIF-1α degradation and activating hypoxia-like signalling. (B) The three enzyme catalysed reactions that can convert l-amino acids to a-keto acids.

In 2016, Teng et al. discovered that mouse LDH-C, besides catalysing pyruvate-lactate interconversion, also catalyses α-KG reduction to s-2-hydroxyglutarate (s-2-HG). They observed s-2-HG levels in normal mouse testes were over10-fold higher than in other tissues, attributing this to LDH-C activity. However, they concluded human LDH-C could not catalyse this reaction at neutral pH^73^. In contrast, Tan et al. demonstrated that human LDH-C can catalyse α-KG to s-2-HG conversion at pH 7.4. Comparing catalytic efficiencies using α-KG as substrate revealed LDH-C exhibits the strongest activity among the human isozymes^15^. Teng et al. conclude that human LDH-C only produce lactate based on the very low *K*_cat_ value for α-KG. However, according to their data, they neglect that human LDH-C show more than 2-fold *K*_cat_ than human LDH-A. This is consistent with Tan’s data. Moreover, Intlekofer et al. also show that human LDH-C can convert α-KG to s-2-HG^74^. α-KG, a central TCA cycle metabolite, critically regulates cancer metabolism, impacting energy production, epigenetic regulation, redox homeostasis, and oncogenic signalling (Figure 4A). Under nutrient deprivation (e.g., low glucose), cancer cells utilise glutamine-derived α-KG to replenish TCA intermediates, enabling OXPHOS and supporting nucleotide/lipid biosynthesis^26^. Beyond metabolism, α-KG serves as an essential cofactor for chromatin-modifying enzymes: TET dioxygenases (catalysing DNA demethylation)^75^ and JmjC-domain histone demethylases^76^. These modifications can activate oncogenes or silence tumour suppressors. Furthermore, α-KG-derived glutamate is a precursor for glutathione (GSH) synthesis, neutralising ROS and protecting against oxidative damage^77^. α-KG also modulates oncogenic signalling: (1) Under hypoxia, reduced α-KG impairs prolyl hydroxylase domain (PHD) activity, stabilising hypoxia-inducible factor 1α (HIF-1α) and activating hypoxia-responsive genes promoting angiogenesis and metastasis^78^^,^^79^. (2) α-KG enhances mTORC1 signalling by facilitating Rag GTPase-mediated lysosomal localisation, driving cell growth and proliferation^80^.

S-2-HG, a structural analog of α-KG generated by LDH-C-catalysed reduction, plays a pivotal oncogenic role. Its structural similarity allows it to competitively inhibit 2-oxoglutarate (2OG)-dependent dioxygenases. First, it disrupts epigenetic regulation by suppressing TET proteins and JmjC histone demethylases, leading to DNA and histone hypermethylation. Second, S-2-HG inhibits PHD2, stabilising HIF-1α and amplifying HIF-driven transcription even under normoxia. These mechanisms drive metabolic reprogramming and tumorigenesis via sustained HIF-1α activation and mTOR pathway dysregulation^25^.

Tan et al.^15^ demonstrated that human LDH-C catalyses the interconversion of α-KG and s-2-HG, as well as oxaloacetate (OAA) and malate (unpublished data). α-Keto acids like α-KG and OAA are critical TCA cycle intermediates (Figure 4A) and are closely linked to amino acid metabolism, as many amino acids convert to α-keto acids via single-step reactions (Figure 4B). LDH-C’s broad substrate specificity, conserved in mice and humans, implies a significant, previously unrecognised role in regulating these pathways.

## 6. Concluding remarks and future directions

Different LDH isozymes are highly or aberrantly expressed across various cancer types, with expression levels varying among malignancies. Overexpression generally promotes cancer cell proliferation, while knockdown inhibits growth, suggesting functional similarities for LDH-A, LDH-B, and LDH-C in tumour biology.

The prevalent view that LDH-A catalyses pyruvate-to-lactate conversion while LDH-B catalyses the reverse reaction is overly simplistic and potentially misleading. This dichotomy may restrict cancer research by overemphasising one isozyme while overlooking others. Biochemically, all three isozymes catalyse the interconversion of pyruvate and lactate. Consequently, all contribute to supporting cancer metabolism by facilitating glycolytic energy production and supplying anabolic precursors.

Given the overlapping and potentially complementary functions of the three isozymes, comprehensively investigating their expression patterns and regulatory mechanisms is essential. Although several studies address individual LDH isozyme regulation^27^^,^^40^, the underlying mechanisms remain incompletely understood. Moreover, few studies concurrently analyse all three LDH isozymes’ expression and regulation within a single cancer type. A critical unresolved question is why different isozymes display distinct expression profiles across cancers and what molecular events drive their dysregulation in specific tumour contexts.

LDH-C, as a CTA, presents an attractive target, especially for patients not concerned with fertility preservation. Owing to its distinct structural characteristics compared to LDH-A and LDH-B^15^, LDH-C may be more amenable to selective inhibition. Therefore, targeting aberrantly expressed LDH-C in tumours represents a promising direction for future therapeutic development.

Inhibition of lactate dehydrogenase A (LDH-A) has emerged as a promising strategy to alleviate immunosuppression within the tumour microenvironment (TME) and enhance the efficacy of cancer immunotherapy. Accumulating evidence demonstrates that both pharmacological and genetic inhibition of LDH-A can synergise with immune checkpoint inhibitors to improve antitumor immune responses^81–83^. In contrast, no published studies have directly investigated the combination of LDH-B or LDH-C inhibition with immunotherapy. Given the distinct properties of LDH-B and LDH-C (Table 1), exploring their isoform-specific inhibition in combination with immunotherapeutic approaches represents a compelling and underexplored direction. Furthermore, due to the pronounced metabolic plasticity of cancer cells, effective combination strategies will likely need to extend beyond glycolytic inhibition to concurrently target additional metabolic pathways.

## Data Availability

No datasets were generated or analysed during the current study.

## Abbreviations

LDH: Lactate dehydrogenase
CTA: Cancer/testis antigen
α-KG: α-Ketoglutarate
EMT: Epithelial–mesenchymal transition
OXPHOS: Oxidative phosphoryla­tion
ROS: Reactive oxygen species
mLDH: Mitochondrial LDH
mLOC: Mitochondrial lactate oxidation complex
mMCT1: Mitochondrial monocarboxylate transporter 1
COx: Cytochrome oxidase
acetyl-CoA: Acetyl coenzyme A
TCA: Tricarboxylic acid cycle
DDR: DNA damage response
s-2-HG: S-2-hydroxyglutarate
HIF: Hypoxia-inducible factor
mTOR: Mechanistic target of rapamycin
TET: Ten-eleven translocation
GSH: Glutathione
PHD: Prolyl hydroxylase domain
OAA: Oxaloacetate
